# Increasing system-wide implementation of opioid prescribing guidelines in primary care: findings from a non-randomized stepped-wedge quality improvement project

**DOI:** 10.1186/s12875-020-01320-9

**Published:** 2020-11-28

**Authors:** Aleksandra E. Zgierska, James M. Robinson, Robert P. Lennon, Paul D. Smith, Kate Nisbet, Mary W. Ales, Deanne Boss, Wen-Jan Tuan, Regina M. Vidaver, David L. Hahn

**Affiliations:** 1 Departments of Family and Community Medicine, Public Health Sciences, and Anesthesiology and Perioperative Medicine, Penn State College of Medicine, 500 University Drive, PA 17033 Hershey, USA; 2 Center for Health Systems Research and Analysis, University of Wisconsin-Madison, 1109C WARF Building, 610 Walnut Street, Madison, WI 53726 USA; 3 Department of Family and Community Medicine, Penn State College of Medicine, 500 University Drive, Hershey, PA 17033 USA; 4 Department of Family Medicine and Community Health, Wisconsin Research and Education Network (WREN), University of Wisconsin-Madison, School of Medicine and Public Health, 1100 Delaplaine Court, Madison, WI 53715 USA; 5 Interstate Postgraduate Medical Association, P.O. Box 5474, Madison, WI 53705 USA; 6 Department of Family Medicine and Community Health, University of Wisconsin-Madison, School of Medicine and Public Health, 1100 Delaplaine Court, Madison, WI 53715 USA

**Keywords:** Quality improvement, Primary care, Chronic pain, Health care delivery, Physician behavior, Opioids

## Abstract

**Background:**

Clinician utilization of practice guidelines can reduce inappropriate opioid prescribing and harm in chronic non-cancer pain; yet, implementation of “opioid guidelines” is subpar. We hypothesized that a multi-component quality improvement (QI) augmentation of “routine” system-level implementation efforts would increase clinician adherence to the opioid guideline-driven policy recommendations.

**Methods:**

Opioid policy was implemented system-wide in 26 primary care clinics. A convenience sample of 9 clinics received the QI augmentation (one-hour academic detailing; 2 online educational modules; 4–6 monthly one-hour practice facilitation sessions) in this non-randomized stepped-wedge QI project. The QI participants were volunteer clinic staff. The target patient population was adults with chronic non-cancer pain treated with long-term opioids. The outcomes included the clinic-level percentage of target patients with a current treatment agreement (primary outcome), rates of opioid-benzodiazepine co-prescribing, urine drug testing, depression and opioid misuse risk screening, and prescription drug monitoring database check; additional measures included daily morphine-equivalent dose (MED), and the percentages of all target patients and patients prescribed ≥90 mg/day MED. T-test, mixed-regression and stepped-wedge-based analyses evaluated the QI impact, with significance and effect size assessed with two-tailed *p* < 0.05, 95% confidence intervals and/or Cohen’s *d*.

**Results:**

Two-hundred-fifteen QI participants, a subset of clinical staff, received at least one QI component; 1255 patients in the QI and 1632 patients in the 17 comparison clinics were prescribed long-term opioids. At baseline, more QI than comparison clinic patients were screened for depression (8.1% vs 1.1%, *p* = 0.019) and prescribed ≥90 mg/day MED (23.0% vs 15.5%, *p* = 0.038). The stepped-wedge analysis did not show statistically significant changes in outcomes in the QI clinics, when accounting for the comparison clinics’ trends. The Cohen’s *d* values favored the QI clinics in all outcomes except opioid-benzodiazepine co-prescribing. Subgroup analysis showed that patients prescribed ≥90 mg/day MED in the QI compared to comparison clinics improved urine drug screening rates (38.8% vs 19.1%, *p* = 0.02), but not other outcomes (*p* ≥ 0.05).

**Conclusions:**

Augmenting routine policy implementation with targeted QI intervention, delivered to volunteer clinic staff, did not additionally improve clinic-level, opioid guideline-concordant care metrics. However, the observed effect sizes suggested this approach may be effective, especially in higher-risk patients, if broadly implemented.

**Trial registration:**

Not applicable.

## Background

Chronic non-cancer pain is a common, disabling condition, [1] for which opioids have been increasingly prescribed over the past decades, despite a paucity of research on long-term benefits and strong evidence for dose-dependent harm [2, 3], compounded by inappropriate opioid prescribing practices [4, 5]. Implementation of evidence- and guideline-based management of opioid therapy in chronic non-cancer pain can reduce inappropriate prescribing and harm from opioids [4–11]. Adopting guideline-concordant management of opioid therapy in primary care is particularly important because primary care clinicians prescribe approximately half of opioid analgesics [12]. However, research on effective ways for dissemination and implementation of practice guidelines is insufficient, shows mixed results, and does not provide clear guidance on methods for successful adoption of guideline-recommended practices, including in primary care [13–15]. In general, implementation of guidelines has been challenging, with those characterized by high clinical complexity and low research evidence support, as is the case with opioid guidelines [3, 16–19], showing low adoption rates [13, 14].

In February 2016, one academic health system implemented a guideline-driven [16–18] opioid therapy management policy (opioid policy) in primary care via informational sessions for clinicians and clinic teams (routine rollout) [20]. Our project goal was to assess if a multi-faceted, clinic-level quality improvement (QI) intervention in addition to routine rollout, compared to routine rollout alone, would improve guideline- and policy-concordant care and associated metrics [16–18], assessed via de-identified clinic-level electronic health record (EHR) data.

## Methods

This report follows the SQUIRE 2.0 standards for QI reporting [21]. Details on the routine rollout, QI protocol development, components and implementation are described elsewhere [20]. The Institutional Review Board deemed the project a QI initiative, not constituting human subjects research, as defined under 45 CFR 46.102(d).

### Context and setting

The academic health system in Wisconsin, USA included 35 primary care (internal medicine and family medicine) clinics in rural, suburban and urban settings, caring for 200,000 adult primary care patients. In February 2016, it introduced a guideline-driven opioid policy on the management of long-term (≥ 3 months) opioid therapy in primary care adult outpatients with chronic non-cancer pain (“target patients”). The opioid policy provided multiple guideline-concordant recommendations for safety, treatment response monitoring, and management in this target population [20]. The system-wide routine rollout of this policy first involved pilot-testing by the health system of the rollout methods with 3 clinics in the fall of 2015, prior to the system-wide rollout in February 2016 [20]. The health system’s policy rollout consisted of an one-hour in-person meeting for clinicians; a one-hour online training session for clinic staff; and two follow-up tele-meetings to address comments/questions from the clinic staff [20]. The health system’s leadership approved this QI project to determine if augmenting the system’s routine rollout would improve patient outcomes.

### Clinic selection

The health system included 35 primary care clinics. Nine of these clinics were involved in other opioid QI initiatives and were excluded from the recruitment pool, yielding 26 clinics eligible for the QI project and included in the analyses. Among these 26 clinics, those with the highest number of target patients were approached first. Three of the approached clinics declined (“too busy for new QI efforts”). The first 9 consenting clinics (convenience sample) were enrolled into a non-randomized stepped-wedge QI project. The QI intervention was initiated immediately after the health system’s routine policy rollout in February 2016. It was delivered over 4–6 months at each clinic and implemented in three waves (March–July 2016; September–December 2016; January–June 2017), with 3 clinics per wave; the QI wave assignment was non-random, based on each clinic’s preference. The non-random approach to both clinic selection and the timing of each clinic’s participation in the QI intervention resulted from the health system and clinics’ requests to allow maximum flexibility for each clinic’s involvement.

### Participants

The QI participants were volunteer clinical staff (prescribers, nurses and others) at each intervention clinic. The evaluation subjects (target patient population) were identified by the search of EHR-based data from the problem list, encounter, and billing records, using the health system*-*developed criteria*:* age ≥ 18 years old; active-patient status (seen at the clinic in the past 3 years); primary care provider within the health system; no diagnosis of malignant neoplasm (except non-melanoma skin cancer) or palliative or hospice care status; and meeting at least one of the two criteria: 1) ≥1 opioid prescription issued in the prior 45 days and ≥ 3 opioid prescriptions issued in the prior 4 months; or 2) ≥1 opioid prescription issued in the prior 45 days, and presence of a chronic pain diagnosis and a controlled substance agreement. For the analyses, buprenorphine was excluded from the “eligible opioid” list due to its primary utility locally as a treatment for opioid use disorder.

### QI intervention

The intervention was developed by the project’s multidisciplinary team, following the recommended approach to QI in primary care [15], and with input from the health system leadership and clinicians to ensure alignment with opioid prescribing guidelines and the health system’s policies, procedures and resources, as detailed elsewhere [20]. Briefly, the QI intervention at each clinic consisted of: a) one 1-h academic detailing session, delivered by the project physicians, outlining the project, the national guidelines, and the health system’s opioid policy recommendations; b) two 20–21 question online educational modules: one on shared decision-making in the context of opioid therapy for chronic pain, and another on the guideline and health system policy recommendations for opioid therapy management; and c) six 1-h practice facilitation (PF) sessions delivered at each clinic over 4–6 months by the project’s trained facilitators. The PF sessions focused on optimizing clinical workflows to promote clinician adherence to the guideline and health system policy recommendations with measurable outcomes (“QI targets”). The selection of QI targets for the PF sessions was driven by each clinic team’s preference [20]. Participating clinical staff were eligible for up to 23 *American Medical Association Physician’s Recognition Award Category 1™ Credits* for completing the intervention.

### Measures

#### Process measures

Quantitative and qualitative process or explanatory measures related to the QI intervention were collected to better understand the processes underlying the hypothesized change in the main outcomes. They included attendance and completion rates of the intervention components, pre-post intervention surveys of the participating clinicians/staff about their confidence, attitudes, barriers and facilitators toward the recommended management of patients with opioid-treated chronic pain, and surveys of participating clinicians/staff about the intervention components.

#### Outcome measures

Were extracted and assessed monthly from the EHR (Epic Systems Corporation) from baseline (January 2016) through project end (December 2017). De-identified EHR data were analyzed at the clinic level. Outcome measures were selected based on the opioid prescribing guideline [16–18] and policy recommendations, and availability of the corresponding EHR data entered as a part of the health system’s routine care [20]. The percentage of target patients with a “current” (signed within the past 12 months, and assessed monthly) treatment agreement was chosen as the primary outcome based on the health system’s opioid policy, which recommended its routine completion, followed by annual updates*.*^20^ Secondary outcome measures were: a) “current” urine drug testing (UDT) and b) “current” depression screen with a two- or nine-item Patient Health Questionnaire (PHQ) [22, 23] (a positive screen using a two-item questionnaire automatically triggered completion of a nine-item questionnaire); c) “current” prescription drug monitoring program (PDMP) database check; d) completion of the opioid misuse risk screen with the Diagnosis, Intractability, Risk, Efficacy (D.I.R.E.) tool [24]; and e) the rate of opioid-benzodiazepine co-prescribing in at least one of the past 3 months. Secondary outcomes a-d were based on the health system’s policy. The co-prescribing measure was not a part of the health system’s policy, but was included based on guideline recommendations advising against co-prescribing [16–18]. Additional measures of interest commonly used to assess opioid interventions [15–18] included: a) percentage of target patients relative to the total adult clinic panel; b) daily opioid dose, calculated per target patient as an average morphine-equivalent dose (MED; milligrams/day) by adding up the doses of all opioids (except buprenorphine) prescribed for outpatient treatment in the prior 90 days and dividing the sum by 90; and c) percentage of target patients prescribed MED ≥90 mg/day (past 90 days). Opioid and benzodiazepine prescription data were extracted from the medication list.

### Statistical analysis

Nine clinics were predicted to provide 80% power to detect a clinically meaningful (20%) increase in the use of a treatment agreement (see Additional File 1 for a detailed sample size discussion). Primary and secondary outcomes were defined a priori. SAS® Version 9.4 was used for statistical analyses. Baseline (January 2016) and project end (December 2017) data were collected using clinic-level averages, weighted by target patient panel size per clinic, so that the change in the clinic-level variables was independent of the clinic’s target patient panel size. Descriptive statistics were used to describe these data, using numbers (percentages), and means with standard deviation (SD) or standard error (SE). Single and two-sample means tests evaluated outcome changes between baseline and exit data within and between the intervention and comparison clinics, respectively. For the PDMP outcome measure, the end date was changed from December to March 2017 due to changes in state law and health system requirements, which led to approximately 100% PDMP check documentation across the clinics starting in April 2017. The primary evaluation of intervention impact was conducted using a mixed-effects regression analysis model. By identifying the three distinct project periods (pre-, during, and post-intervention) for each QI clinic, we were able to evaluate and contrast these distinct, specific periods across the project duration. Therefore, the model leveraged the monthly EHR data and accounted for the timing of intervention delivery in the QI clinics by contrasting clinic-level pre-intervention data, with data collected during and then after the intervention (stepped-wedge analysis). The stepped-wedge analysis was further augmented by adding comparison clinics’ monthly data during the same assessment period. Linear curves were fitted to the monthly outcomes as fixed effects, with baseline values and slopes of change separately estimated for QI intervention and comparison clinics. Additional fixed effects were included to allow the slopes of fitted curves for intervention clinic outcomes to change in relation to the intervention and post-intervention periods. Random effects were included at the levels of both the primary care provider (PCP) within each clinic and the clinic as a whole to account for correlation among monthly observations from the same PCP or the same clinic. Observations were also weighted by the number of target patients within each PCP’s monthly panel. Estimates of the differential slopes (pre-intervention, intervention and post-intervention) for the QI clinics and a single, study-long slope for the comparison clinics were used to assess the specific impact of the intervention on the QI clinics’ outcomes. Baseline differences between QI and comparison clinic characteristics, and between clinic and PCPs within each study group, were accounted for in differential baseline intercepts and slopes, and with random intercept and slope effects, respectively. See Additional File 2 for details of the mixed effect model and result interpretation.

A subgroup analysis was conducted among target patients treated with MED ≥90 mg/day, because of their increased risk for opioid-related harm [18].

The significance and magnitude of changes were assessed with *p* values (significance level: two-tailed *p* < 0.05), 95% Confidence Intervals (CIs), and/or Cohen’s *d* effect size (ES, 0.2–0.4: small; 0.5–0.7: medium; ≥0.8: large) [25].

## Results

### Process-related findings

A total of 215 unique health care providers, including 73 prescribers and 142 other clinic staff from the enrolled 4 family medicine and 5 internal medicine clinics completed at least one component of the QI intervention (QI participants; Table 1). Among the QI participants, 48.4% completed half or more of the intervention components; 44.7% completed at least 4 of the 6 in-person practice facilitation sessions; 31.2% completed the opioid prescribing and 23.2% completed the shared decision making online modules (Table 1). The intervention participation was voluntary, and not all clinic health care providers participated; although data on the total clinic staff were not collected or available, it was estimated that fewer than 50% of each clinic’s staff received the intervention.

**Table 1:** Completion of the intervention components among the quality improvement (QI) intervention clinics’ staff

Position	Completed ≥1 intervention component	Completed ≥50% of the intervention components^a^	Completed Academic Detailing	Completed Online Opioid Module	Completed Online Shared Decision Making Module	Completed ≥1 Practice Facilitation Session	Completed ≥4 Practice Facilitation Sessions
**WAVE 1 INTERVENTION CLINICS**							
**Clinic 1: Internal Medicine (urban, residency clinic)**							
MD, DO, #	7	5	6	4	3	6	2
NP, PA, #	3	0	1	1	0	3	1
RN, MA, CMA, LPN, #	17	9	1	10	4	16	10
Other staff, #	7	1	0	1	0	1	1
Total, #	34	15	8	16	7	26	14
**Clinic 2: Internal Medicine (urban, non-residency clinic)**							
MD, DO, #	8	5	7	5	4	1	1
NP, PA, #	0	0	0	0	0	0	0
RN, MA, CMA, LPN, #	24	14	23	11	9	6	6
Other staff, #	8	2	4	2	2	2	2
Total, #	40	21	34	18	15	9	9
**Clinic 3: Family Medicine (rural; residency clinic)**							
MD, DO, #	8	3	6	3	3	5	2
NP, PA, #	1	1	1	1	1	1	1
RN, MA, CMA, LPN, #	9	8	8	2	2	8	8
Other staff, #	4	1	3	0	0	5	2
Total, #	22	13	18	6	6	19	13
**WAVE 2 INTERVENTION CLINICS**							
**Clinic 4: Family Medicine (urban; residency clinic)**							
MD, DO, #	4	3	3	2	1	4	3
NP, PA, #	1	0	0	0	0	1	0
RN, MA, CMA, LPN, #	6	3	3	4	4	5	5
Other staff, #	3	2	3	1	1	3	2
Total, #	14	9	9	7	6	13	10
**Clinic 5: Family Medicine (urban; residency clinic)**							
MD, DO, #	11	5	9	4	2	4	4
NP, PA, #	4	1	3	0	0	2	1
RN, MA, CMA, LPN, #	11	3	10	3	3	3	2
Other staff, #	8	0	0	0	0	5	4
Total, #	34	9	22	7	5	14	11
**Clinic 6: Internal Medicine (urban; residency clinic)**							
MD, DO, #	7	1	7	0	0	6	2
NP, PA, #	3	1	3	1	0	3	1
RN, MA, CMA, LPN, #	14	5	12	2	1	14	5
Other staff, #	3	0	0	0	0	2	0
Total, #	27	7	22	3	1	25	8
**WAVE 3 INTERVENTION CLINICS**							
**Clinic 7: Family Medicine (suburban; non-residency clinic)**							
MD, DO, #	3	3	2	1	1	3	3
NP, PA, #	1	1	1	1	1	1	0
RN, MA, CMA, LPN, #	7	5	6	2	2	6	5
Other staff, #	2	0	0	0	0	2	1
Total, #	13	9	9	4	4	12	9
**Clinic 8: Internal Medicine (urban; residency clinic)**							
MD, DO, #	4	1	3	1	1	2	1
NP, PA, #	2	2	2	1	1	1	1
RN, MA, CMA, LPN, #	6	4	5	0	0	6	4
Other staff, #	3	3	2	1	0	3	3
Total, #	15	10	12	3	2	12	9
**Clinic 9: Internal Medicine (urban; residency clinic)**							
MD, DO, #	4	4	4	2	1	4	3
NP, PA, #	2	1	2	0	1	1	0
RN, MA, CMA, LPN, #	10	7	9	1	2	8	7
Other staff, #	0	0	0	0	0	0	0
Total, #	16	12	15	3	4	13	10
**ALL INTERVENTION CLINICS**							
MD, DO, #	56	30	47	22	16	34	22
NP, PA, #	17	7	13	5	4	12	6
RN, MA, CMA, LPN, #	104	58	77	35	27	68	53
Other staff, #	38	9	12	5	3	23	15
**Total, #**	**215**	**104**	**149**	**67**	**50**	**137**	**96**

^a^50% completion applies to clinicians/staff who completed two of the four intervention components: Academic Detailing, Online Opioid Module, Online Shared Decision Making Module, Practice Facilitation (4 of 6 sessions)

*CMA* Certified Medical Assistant, *DO* Doctor of Osteopathic Medicine, *MA* Medical Assistant, *MD* Doctor of Medicine, *PA* Physician Assistant, *NP* Nurse Practitioner, *RN* Registered Nurse

Other process-related findings are detailed in Additional File 4. Briefly, pre-intervention, the clinicians/staff identified responsible opioid prescribing practices, shared decision making, and the management of patients with chronic pain as areas of educational need. Based on the post-intervention evaluation, this need was met through the QI intervention’s components. Overall, the intervention participants identified learning through the project about the conduct of a QI process and its impact assessment, and working better as a team as important outcomes of the project and useful skills that are “transferrable” toward other initiatives.

### Baseline characteristics of the target patient population

Across the 26 evaluated primary care clinics, 3148 target patients (58.1% women, mean age 53.3 (SD 13.8) years) were identified, with 1431 in the QI and 1717 in the comparison clinics. These patients comprised 1.9% of the QI and 2.0% of the comparison clinics’ adult patient panels. A comparison of weighted, clinic-level baseline characteristics showed that the target patients in the QI and comparison clinics (Table 2) did not differ in a statistically significant way in relatively high daily MED doses and the rates of opioid-benzodiazepine co-prescribing. They also did not differ in their overall low rates of “current” treatment agreements, urine drug testing, completed opioid risk assessment, and documented PDMP check. However, the QI clinics, relative to the comparison clinics, had higher rates of completed depression screening (8.1% (SD 10.4) vs. 1.1% (SD 1.3), *p* = 0.019) and of patients prescribed MED ≥90 mg/day (23.0% (SD 8.8) vs. 15.5% (SD 7.3), *p* = 0.038). No statistically significant differences in baseline characteristics were noted in a subgroup of target patients treated with MED ≥90 mg/day in the QI (*N* = 359) and comparison (*N* = 283) clinics.

**Table 2:** Baseline characteristics of the target patient population

Characteristics	Intervention clinics at baseline (*N* = 1431)	Comparison clinics at baseline (*N* = 1717)	*p* value^*^
Women, % (SD)	57.7 (7.1)	60.7 (8.9)	0.400
Mean age, years, mean (SD)	53.6 (3.2)	53.8 (3.5)	0.864
Completed treatment agreement (past 12 months), % (SD)	24.8 (13.8)	29.2 (18.3)	0.531
Completed urine drug testing (past 12 months), % (SD)	24.7 (11.8)	31.3 (16.2)	0.285
Completed depression screening (past 12 months), % (SD)	8.1 (10.4)	1.1 (1.3)	**0.019**
Completed opioid misuse risk assessment, % (SD)	0.2 (0.4)	0.7 (1.8)	0.478
Documented PDMP Check (past 12 months), % (SD)	0.0 (0.1)	0.3 (1.2)	0.503
Co-prescribed benzodiazepines in at least 1 out of 3 past months, % (SD)	19.9 (4.3)	24.7 (7.4)	0.089
Percentage of the adult clinic population, % (SD)	2.0 (0.9)	2.1 (0.6)	0.906
MED (past 90 days), mg/day, mean (SD)	75.7 (29.7)	55.9 (19.4)	0.063
MED ≥ 90 mg/day (past 90 days), % (SD)	23.0 (8.8)	15.5 (7.3)	**0.038**

*Population of adult patients with opioid-treated chronic non-cancer pain in the 9 intervention and 17 comparison primary care clinics: characteristics at baseline (January 2016) based on the equally-weighted clinic averages*

*MED* Morphine-equivalent Dose, *PDMP* Prescription Drug Monitoring Program, *SD* standard deviation

^*^ *p* value was determined using the two-sample means test comparing clinic-level values for intervention versus comparison clinic groups

### Primary outcome analysis: mixed-effects regression analysis

Regression analysis of monthly outcomes by clinic and prescriber recognized variations in the timing of the QI intervention from clinic to clinic within the QI clinic group. This analysis did not reveal statistically significant changes in outcomes in the stepped-wedge pre-post analyses, which contrasted the pre-intervention period with the combined intervention and post-intervention periods. Augmentation with data from the comparison clinics did not impact these findings. See Additional File 3 for detailed results.

However, when the evaluation specifically focused on the intervention period, separating it from the post-intervention period, several statistically significant changes were noted in the QI clinics, after accounting for the trends in the comparison clinics (see Additional File 3). The completion rate of new treatment agreements (incidence rate) increased in the QI clinics during the intervention months both in the overall target population (by 9.4%; *p* = 0.023; 95%CI = [0.028,0.159]) and in the subgroup of patients treated with MED ≥90 mg/day (by 15.9%; *p* = 0.044, 95%CI = [0.029,0.289]); these differences were not sustained post-intervention in either group. In addition, in the QI clinics, among the overall target population and in the high-dose subgroup, MED decreased post-intervention (by − 2.75 and − 6.50 mg/day, respectively), but not during the intervention period (0.80 and 12.43 mg/day, respectively).

### Secondary outcomes: means tests

Both the QI and comparison clinics improved on all outcomes among the target population (Table 3), except the prevalence of opioid-benzodiazepine co-prescribing, which improved in the comparison clinics only (*p* = 0.006). When comparing the change in outcomes between the QI and comparison clinics, no statistically significant differences were noted. However, Cohen *d* effect sizes favored the QI clinics, except for opioid-benzodiazepine co-prescribing (a small ES in the intervention and a moderate ES in the comparison clinics).

**Table 3:** Target Patient Population: Change in Outcomes

Characteristics	Change in Intervention clinics (*N* = 1255 at exit)			Change in Comparison clinics (*N* = 1632 at exit)			Change in Intervention versus Comparison clinics		
	Change from baseline	*p* value^†^	Cohen’s *d*	Change from baseline	*p* value^†^	Cohen’s *d*	Difference	*p* value^‡^	Cohen’s *d*
Completed treatment agreement (past 12 months), % (SE), 95% CI (LB,UB)	32.0 (6.7) (18.8, 45.2)	< 0.001	2.1	29.2 (6.7) (16.1, 42.4)	< 0.001	1.5	2.8 (9.6) (−16.1, 21.6)	0.816	0.1
Completed urine drug testing (past 12 months), % (SE), 95% CI (LB,UB)	33.4 (7.0) (19.8, 47.1)	< 0.001	2.6	25.7 (5.3) (15.3, 36.1)	< 0.001	1.7	7.7 (8.7) (−9.3, 24.7)	0.277	0.4
Completed depression screening (past 12 months), % (SE), 95% CI (LB,UB)	49.2 (6.1) (37.2, 61.1)	< 0.001	3.4	55.4 (6.9) (41.8, 69.0)	< 0.001	3.3	6.2 (9.6) (−25.1, 12.6)	0.526	0.3
Completed opioid misuse risk assessment, % (SE), 95% CI (LB,UB)	5.4 (3.0) (−0.5, 11.3)	0.070	0.9	10.1 (5.8) (−1.4, 21.5)	0.050	0.7	−4.7 (7.4) (−19.2, 9.9)	0.417	0.3
Documented PDMP^*^ Check (past 12 months), % (SE), 95% CI (LB,UB)	50.7 (6.3) (38.3, 63.1)	< 0.001	3.8	39.9 (7.8) (24.6, 55.2)	< 0.001	2.0	10.8 (10.8) (−10.4, 32.0)	0.301	0.4
Co-prescribed benzodiazepines in at least 1 out of 3 past months, % (SE), 95% CI (LB,UB)	−1.2 (1.6) (−4.3, 1.9)	0.461	0.3	−5.4 (1.8) (−9.0, − 1.8)	0.006	0.7	4.3 (2.7) (−0.9, 9.5)	0.143	0.7
Percentage of the adult clinic population, % (SE), 95% CI (LB,UB)	−0.3 (0.2) (− 0.6, 0.0)	0.042	0.4	−0.4 (0.1) (− 0.6, − 0.1)	0.056	0.5	0.0 (0.2) (− 0.3, 0.4)	0.547	0.1
MED (past 90 days), mg/day, mean (SE), 95% CI (LB,UB)	−11.6 (2.4) (− 16.4, − 6.9)	< 0.001	0.4	−9.9 (3.0) (− 15.8, − 4.1)	0.0001	0.6	−1.7 (4.0) (− 9.6, 6.2)	0.937	0.2
MED ≥ 90 mg/day (past 90 days), % (SE), 95% CI (LB,UB)	−3.5 (1.1) (− 5.8, − 1.3)	0.002	0.4	− 1.6 (0.7) (− 3.0, − 0.1)	0.004	0.2	−2.0 (1.3) (− 4.5, 0.6)	0.270	0.7

*Population of adult patients with opioid-treated chronic non-cancer pain in the 9 intervention and 17 comparison primary care clinics; characteristics at exit (December 2017) and their patient-weighted change from baseline*

*CI (LB,UB)* Confidence Interval (Lower Bound, Upper Bound), *MED* Morphine-equivalent Dose, *PDMP* Prescription Drug Monitoring Program, *SE* standard error

^*^ Exit data for the PDMP outcome was collected in March 2017; starting in April 2017, the PDMP check documentation rose to approximately 100% across all clinics due to the changes in documentation requirements

^†^ *p* value was determined using the single sample mean test comparing clinic-level changes in outcomes from baseline to exit

^‡^ *p* value was determined using the two-sample means test comparing clinic-level changes in outcomes from baseline to exit for intervention versus comparison clinic groups

Among the subgroup of target patients prescribed MED ≥90 mg/day (Table 4), all outcomes tended to improve in both the QI and comparison clinics. Comparing the change in outcomes, prevalence of urine drug screening increased twice as much in the QI clinics (38.8% (SE 4.4) vs. 19.1% (SE 7), *p* = 0.020). While there were no other statistically significant differences between the QI and comparison clinics, Cohen’s *d* effect sizes again favored the QI clinics.

**Table 4:** Target Patient Population Treated with ≥90 mg/day of Morphine-equivalent Opioid Dose: Change in Outcomes

Characteristics	Change in Intervention clinics (*N* = 271 at exit)			Change in Comparison clinics (*N* = 248 at exit)			Change in Intervention versus Comparison clinics		
	Change from baseline	*p* value^†^	Cohen’s *d*	Change from baseline	*p* value^†^	Cohen’s *d*	Difference	*p* value^‡^	Cohen’s *d*
Completed treatment agreement (past 12 months), % (SE), 95% CI (LB,UB)	41.1 (9.3) (23.0, 59.3)	< 0.001	1.9	40.9 (8.2) (24.9, 56.9)	< 0.001	2.0	0.2 (12.2) (−23.7, 24.1)	0.783	0.0
Completed urine drug testing (past 12 months), % (SE), 95% CI (LB,UB)	38.8 (4.4) (30.2, 47.4)	< 0.001	2.3	19.1 (7.0) (5.4, 32.8)	0.036	1.0	19.8 (10.4) (−0.7, 40.2)	**0.020**	0.8
Completed depression screening (past 12 months), % (SE), 95% CI (LB,UB)	54.2 (7.6) (39.4, 69.0)	< 0.001	2.6	60.6 (9.7) (41.5, 79.7)	< 0.001	2.6	−6.3 (13.1) (−32.1, 19.4)	0.528	0.2
Completed opioid misuse risk assessment, % (SE), 95% CI (LB,UB)	7.2 (3.8) (−0.3, 14.7)	0.060	0.9	10.3 (6.6) (−2.7, 23.3)	0.074	0.6	−3.1 (8.5) (−19.7, 13.6)	0.582	0.2
Documented PDMP Check (past 12 months)^*^, % (SE), 95% CI (LB,UB)	56.5 (6.9) (43.0, 70.0)	< 0.001	3.8	45.2 (8.0) (29.6, 60.8)	< 0.001	2.3	11.3 (11.3) (−10.8, 33.4)	0.263	0.4
Co-prescribed benzodiazepines in at least 1 out of 3 past months, % (SE), 95% CI (LB,UB)	−5.8 (3.5) (−12.6, 0.9)	0.091	0.5	−6.0 (5.1) (−16.0, 4.0)	0.039	0.4	0.1 (6.7) (−13.0, 13.3)	0.486	0.0
Percentage of the adult clinic population, % (SE), 95% CI (LB,UB)	−0.2 (0.1) (−0.3, 0.0)	0.030	0.4	−0.1 (0.0) (− 0.1, 0.0)	0.031	0.3	−0.1 (0.1) (− 0.2, 0.1)	0.224	0.5
MED (past 90 days), mg/day, mean (SE), 95% CI (LB,UB)	−26.9 (10.0) (−46.5, −7.3)	0.007	0.5	−33.8 (16.5) (−66.2, −1.4)	0.005	0.6	6.9 (21.6) (−35.3, 49.2)	0.356	0.1

Population of adult patients with chronic non-cancer pain treated with opioid dose ≥ 90 morphine-equivalent mg/day in the 9 intervention and 17 comparison primary care clinics; characteristics at exit (December 2017*) and their patient-weighted change from baseline

*CI (LB,UB)* Confidence Interval (Lower Bound, Upper Bound), *MED* Morphine-equivalent Dose, *PDMP* Prescription Drug Monitoring Program, *SE* standard error

^*^ Exit data for the PDMP outcome was collected in March 2017; starting in April 2017, the PDMP check documentation rose to approximately 100% across all clinics due to the changes in documentation requirements

^†^ *p* value was determined using the single-sample mean test comparing clinic-level changes in outcomes from baseline to exit

^‡^ *p* value was determined using the two-sample means test comparing clinic-level changes in outcomes from baseline to exit for intervention versus comparison clinic groups

## Discussion

The goal of this project was to rigorously evaluate if a multi-component, QI augmentation of “routine” system-level implementation of opioid prescribing policy would help increase clinician adherence to the policy recommendations and improve opioid prescribing practices, assessed via clinic-level EHR data. Strengths of this study include its large sample size, breadth of application, pragmatic approach to testing under actual clinical conditions, and conservative approach to outcome analysis conducted on the global clinic level. Those strengths, however, also led to the primary limitations of this QI project, which did not account for the potential confounders, such as differences between the QI clinics in their staff engagement (percentage of each clinic staff participating in the QI initiative; completion by the participating staff of the intervention components), leadership’s support or the selected improvement targets at each clinic. Not all prescribers received the intervention; yet, because all target patients within a clinic were evaluated as an aggregate EHR data set, we were unable to separate the specific outcomes of patients whose prescribers and other health care providers received the intervention from patients whose clinicians did not receive it. In addition, the participation numbers indicate that prescribers were less engaged in the QI intervention than other clinical staff. These limitations though make all the more remarkable the statistically significant difference in urine drug screening among higher-risk patients (treated with high-dose opioids) and the overall favoring by Cohen’s *d* effect sizes of the change in outcomes in the QI clinics, relative to the comparison clinics.

Another limitation was the absence of randomization in clinic assignment to intervention versus comparison groups. Randomization would have avoided potential selection bias. However, because clinic participation was voluntary, a convenience sample approach was necessary. Although we excluded clinics actively engaged in other opioid-related QI initiatives, clinics we approached that declined to participate may have done so because they had more fully embraced the concept of implementing the system-wide policy changes, such that their addition to the comparison group artificially elevated that cohorts’ average changes. In addition, each intervention clinic selected their own targets for focused improvements, potentially further diluting the intervention effects on any one of the measures.

State-wide legislative changes during our QI project represented an additional potential confounder, which could have substantially impacted clinician behavior [26]. In April 2017, a state law went into effect requiring clinicians to check a patient’s PDMP record before prescribing any controlled substances, including opioids. As a result, many health systems across the state, including the evaluated health system, implemented a system-wide requirement and related workflows to document PDMP check prior to issuing prescriptions for controlled substances. This led to an essentially 100% adherence to this practice starting in April 2017. In addition, in 2017, the State Medical Examining Board introduced a new requirement that all prescribers complete 2 h of approved Continuing Medical Education (CME) on opioid prescribing guidelines for chronic pain. While we ameliorated the impact on our PDMP metric by changing that measure’s end date to March 2017, there is little question that these two external factors and the general increase in public discourse relating to opioid-related harms during the QI period likely contributed to a dilution of our intervention effects. The legal and licensing changes, and the PDMP check requirements can lead to a decrease in the number of prescriptions for opioids and prescribed opioid doses [24]. However, these changes do not necessarily translate into reduction in overdose admission rates, which have in fact increased in the state [25]. This suggest that legislative and other system-level environmental changes with “coercive” components (e.g., potential for legal or disciplinary consequences in the absence of PDMP check documentation prior to providing a prescription for opioids) can alter clinician behavior, a hypothesis supported by the behavior change framework developed by Michie et al. [26].

Our baseline and exit data demonstrated that there was substantial room for improvement in the monitoring of patients treated with long-term opioids, indicating that developing methods to increase clinician adherence to opioid prescribing guidelines remains an important area of research to improve patient outcomes. This QI project did not show statistically significant impact of our intervention on the clinic-level primary or secondary outcomes in a non-randomized stepped-wedge analysis. However, the intervention did yield a marked, statistically significant increase in urine drug screening rate among higher-risk patients treated with high-dose opioids. It also yielded a transient increase in the incidence of new treatment agreements during the intervention period. Further, the magnitude of change in outcomes for all target patients, as assessed by Cohen’s *d* effect size values, suggests that the intervention may help reduce high-dose opioid prescribing in primary care patients treated with long-term opioids for chronic pain. In addition, the QI intervention was well-received and rated as useful by the participating clinicians and other clinic staff, with completion rates suggesting though that in-person PF sessions may have higher utility compared to the self-directed, online educational modules. Intervention participants also identified learning how to formally conduct the QI initiative and assess its impact, and working better as a team as important outcomes of the project and skills that are “transferrable” toward other, future QI initiatives. Primary care clinics with QI orientation and skills are more likely to continue working toward improving their care delivery and patient outcomes [15]. For these reasons, our results offer the chance of conclusive success through a similar intervention, favoring in-person delivery with a broader, entire-clinic implementation, tested under more rigorous conditions, for example, by randomly assigning clinics to the intervention arm and applying a QI intervention to all clinic prescribers of moderate-dose and high-dose opioid-treated patients or evaluating the results only for patients whose prescriber(s) received the intervention.

Both guideline content and construct can impact implementation [27]. Complex guidelines with low “implementability” characteristics, such as the opioid prescribing guidelines and the recommendations targeted by this QI project, have a reduced likelihood of successful implementation [27]. Revising the opioid prescribing guideline with attention to the implementability of recommendations would likely increase its adoption.

## Conclusions

Augmenting routine policy implementation with targeted QI intervention, delivered to volunteer clinic staff, did not additionally improve clinic-level, opioid guideline-concordant care metrics. However, the observed effect sizes suggested this approach may be effective, especially in higher-risk patients, if broadly implemented.

## Supplementary Information

**Additional file 1.** Sample Size Estimation

**Additional file 2.** Mixed effect model and sample results

**Additional file 3.** Summary of mixed effects model results

**Additional file 4.** Process measures and related findings

## Data Availability

The datasets generated and/or analyzed during the current study are not publicly available due to institutional policy on patient data sets, but may be available from the corresponding author on reasonable request.

## Abbreviations

CI: Confidence interval
EHR: Electronic Health Record
LB, UB: Lower bound, upper bound
MED: Morphine equivalent dose
MEDD: Morphine-equivalent daily dose
PC: Primary care
PCP: Primary care provider
PDMP: Prescription drug monitoring program
PF: Practice facilitation
QI: Quality Improvement
SAS: Statistical analysis system
UDT: Urine drug testing
