# Supplementary material for: Increasing system-wide implementation of opioid prescribing guidelines in primary care: findings from a non-randomized stepped-wedge quality improvement project
Source: BMC Fam Pract. 2020 Nov 28;21:245. doi: 10.1186/s12875-020-01320-9 (PMC7700706; doi:10.1186/s12875-020-01320-9)
Supplement: Supplementary file 1 — Additional file 1. Sample Size Estimation [file 12875_2020_1320_MOESM1_ESM.docx]

**Additional File 1. Sample Size Estimation**

A total of nine similar-size primary care clinics were planned to be recruited from among teaching (residency) and community (non-residency) health system clinics in urban, suburban and rural areas. Based on the analysis of preliminary data from the health system’s electronic health record and using conservative estimates for the average adult clinic population (5,000 per clinic) and the proportion of adult patients receiving long-term opioids for chronic pain (3%), we estimated to have greater than 80% power to detect a 20% relative increase in use of a pain medication agreement (primary outcome), assuming that the baseline use at 59% (i.e., an increase from 59% to 120% x 59% = 71%). This power calculation was derived from a traditional cluster randomized trial methodology with an intra-class correlation coefficient of 1.5% and a patient-level standard deviation for the change in outcome of 81%, yielding 84% power with greater than 95% confidence. A stepped-wedge study design will allow each participating clinic to serve as a ‘control’ clinic pre-intervention and a ‘study’ clinic post-intervention, contributing to the fact that stepped wedge designs are likely to be more powerful than comparable traditional CRTs.^1^ Therefore, the proposed approach was estimated to be adequately powered to detect what we considered a clinically meaningful effect of a 20% increase in the percentage of “current” treatment agreement in the target population of adult patients with opioid-treated chronic non-cancer pain.

References:

1. Woertman W, de Hoop E, Moerbeek M, Zuidema SU, Gerritsen DL, [Teerenstra](https://pubmed.ncbi.nlm.nih.gov/?term=Teerenstra+S&cauthor_id=23523551) S. Stepped wedge designs could reduce the required sample size in cluster randomized trials. J Clin Epidemiol, 2013;66(7):752-8. doi: 10.1016/j.jclinepi.2013.01.009
