# Supplementary material for: Increasing system-wide implementation of opioid prescribing guidelines in primary care: findings from a non-randomized stepped-wedge quality improvement project
Source: BMC Fam Pract. 2020 Nov 28;21:245. doi: 10.1186/s12875-020-01320-9 (PMC7700706; doi:10.1186/s12875-020-01320-9)
Supplement: Supplementary file 2 — Additional file 2. Mixed effect model and sample results [file 12875_2020_1320_MOESM2_ESM.docx]

**Additional File 2. Mixed effect model and sample results**

Mixed effect regression modeling was employed to estimate baseline and intervention trends and effects (i.e., the fixed effect components), while also recognizing the correlation between observations from the same prescriber or the same clinic (i.e., the random effect components), following the considerations on modeling clustered data outlined in the recent review article.^1^

The model fixed effect components are piecewise-linear functions describing distinct baseline trends for intervention and comparison clinics, as well as intervention and post-intervention incremental effects for the intervention clinics. Random effects allow the baseline trends and intervention effects to vary by prescriber and clinic, and accounting for correlation among repeated monthly observations from the same prescriber or the same clinic.

For an intervention clinic, we have:

*Y_ipt_ = [a + b t + c t_1_ + d t_2_] + [u_ai_ + u_bi_ t + u_ci_ t_1_ + u_di_ t_2_] + [v_ap_ + v_bp_ t + v_cp_ t_1_ + v_dp_ t_2_] + e_ipt_*

where,

*Y_ipt_* denotes the observed outcome for prescriber *p* within clinic *i* during the study month *t*;

*a + b t + c t_1_ + d t_2_* is the fixed effect shared by each intervention clinic,

*a* is the fitted value of the outcome for the 9 intervention clinics at the beginning of January 2016,

*b* is the fitted monthly change in outcome for the intervention clinics,

*t* is the study month *t* (*t*=1 for the end of January 2016),

*c* is the fitted change to the outcome for the intervention clinics for the entire intervention period,

*t_1_* is the percentage of the intervention period completed,

*d* is the fitted change to the outcome for the intervention clinics for the 6 months following the intervention, and,

*t_2_* is the percentage of the 6-month post-intervention period completed;

*u_ai_ + u_bi_ t + u_ci_ t_1_ + u_di_ t_2_* is the corresponding random effect related to clinic *i*,

*u_ai_, u_bi_,, u_ci_* and *u_di_* are random deviations (with mean zero) from the fixed effect coefficients associated with clinic *i*,

*v_ap_ + v_bp_ t + v_cp_ t_1_ + v_dp_ t_2_* is the corresponding random effect related to prescriber *p*,

*v_ap_, v_bp_,, v_cp_* and *v_dp_* are random deviations (with mean zero) from the fixed effect coefficients associated with prescriber *p*, and,

*e_ipt_* is a random noise term for clinic *i*, prescriber *p* in month *t*.

For a comparison clinic, the intervention effects were removed and adjustments to the baseline intercept and slope were added:

*Y_ipt_ = [a + a* + b t + b* t] + [u_ai_ + u_bi_ t] + [v_ap_ + v_bp_ t] + e_ipt_*,

where,

*a** is the fitted value of the outcome for the comparison clinics minus the fitted value for intervention clinics at the beginning of January 2016 (t=0), and,

*b** is the fitted monthly change in outcome for the comparison clinics minus the slope for the enrolled clinics.

The model parameters were fit weighting each monthly observation by the number of target patients (evaluation subjects) associated with the observation.

The following Additional File 2 Table 1 (Table AF2.1) summarizes the fixed effect results for the primary outcome measure reflecting the percent of target patient population with a “current” treatment agreement. The expected value of the model random effects are zero by definition, so that the estimated fixed effects shown in the table describe the predicted results from the fitted model.

| **Table AF2.1. Fixed Effects Model: Fraction of Target Patient Population with "Current" Treatment Agreement (Signed in the Past 12 Months).** | | | | | | |
| --- | --- | --- | --- | --- | --- | --- |
| **Effect** | **Estimate** | **Standard Error** | **95% CI Lower Bound** | **95% CI Upper Bound** | **t Value** | **Pr > \|t\|** |
| a = Intercept - Intervention clinics | 0.308 | 0.069 | 0.173 | 0.442 | 4.5 | <0.001 |
| a* = Intercept adjust. – Comparison clinics | 0.158 | 0.087 | -0.013 | 0.328 | 1.8 | 0.070 |
| b = Slope - Intervention Clinics | 0.008 | 0.006 | -0.003 | 0.019 | 1.5 | 0.143 |
| b* = Slope adjust. – Comparison clinics | 0.001 | 0.007 | -0.012 | 0.015 | 0.2 | 0.840 |
| c = Intervention Effect | 0.176 | 0.115 | -0.049 | 0.401 | 1.5 | 0.163 |
| d = Post-Intervention Effect | -0.121 | 0.095 | -0.308 | 0.066 | -1.3 | 0.241 |
| Intervention vs. Comparison Clinics | 0.021 | 0.219 | -0.408 | 0.451 | 0.1 | 0.925 |
| Intervention Clinics - Pre vs. Post Intervention | 0.055 | 0.149 | -0.237 | 0.347 | 0.4 | 0.722 |

Table AF2.1 findings indicated that, at baseline, the comparison clinics, compared to the intervention clinics, had a 15.8% higher percentage (p=0.070) of the target population patients who had a “current” treatment agreement (signed within the past 12 months). While the remaining fixed effects were not statistically significant (i.e., p=0.07), it is nevertheless informative to observe the best estimates of the baseline trends and intervention effects as shown in Figure AF2.1. The last two rows of Table AF2.1 represent two aspects of the fitted trends. “Intervention vs. Comparison Clinics” represents the difference in the rates of “current” treatment agreement over the project period between the intervention and comparison clinics, as illustrated in Figure AF2.1, where the solid line indicates the intervention and in the dashed line indicates the comparison clinics’ changes. Both clinic groups improved treatment agreement rates, with the intervention clinics improving by 2.1% more (p=0.93). Finally, “Intervention Clinics: Pre vs. Post Intervention” estimates the net-intervention effect, beyond the pre-intervention baseline trend, within the intervention clinics (the difference between the solid and dotted lines at the end of the study period, Figure AF2.1), indicating an incremental increase in the “current” treatment agreement rate by 5.5% (p=0.72).

**Figure AF2.1.**  **Fixed Effects Model: Change Over Time in the Fraction of Target Patient Population with “Current” Treatment Agreement (Signed in the Past 12 Months).**

The dashed line represents the progress of comparison clinics at increasing the rates of the presence of “current” treatment agreement among the target population patients over the course of the project (Figure AF2.1). The dotted line represents the pre-intervention baseline trend in the “current” treatment agreement rate exhibited by the intervention clinics, which started at a lower baseline rate (by 15.8%; p=0.070) than the comparison clinics. Finally, the solid line represents the monthly rate of “current” treatment agreements in the intervention clinics during the QI intervention (from January to June 2017 in this example) and during the six months following the intervention period. While this example represents only one of the three intervention waves (i.e., 3 intervention clinics), all three waves have the same estimated 6-month intervention and 6-month post-intervention effects. The waves only differ in the time period when these effects occur during the two-year project. The solid line indicates that the intervention clinic rates increased during the QI intervention, approaching the rates of the comparison clinics; but lost most of that relative gain in the post-intervention period.

Table AF2.2 presents a second example of the mixed effect model. In this case, the measure of interest was the average morphine-equivalent daily dose (MEDD) of prescribed opioids in the target population. The corresponding fixed effect trend lines are shown in Figure AF2.2 below.

| **Table AF2.2. Fixed Effects Model: Average Morphine-Equivalent Dose (mg/day) Prescribed per each Target Patient.** | | | | | | |
| --- | --- | --- | --- | --- | --- | --- |
| **Effect** | **Estimate** | **Standard Error** | **95% CI Lower Bound** | **95% CI Upper Bound** | **t Value** | **Pr > \|t\|** |
| a = Intercept - Intervention clinics | 79.253 | 7.659 | 64.242 | 94.264 | 10.4 | <0.001 |
| a* = Intercept adjust. – Comparison clinics | -24.926 | 9.400 | -43.349 | -6.503 | -2.7 | 0.008 |
| b = Slope - Intervention Clinics | -0.532 | 0.183 | -0.890 | -0.175 | -2.9 | 0.007 |
| b* = Slope adjust. – Comparison clinics | 0.117 | 0.221 | -0.316 | 0.550 | 0.5 | 0.596 |
| c = Intervention Effect | 0.801 | 1.106 | -1.367 | 2.969 | 0.7 | 0.469 |
| d = Post-Intervention Effect | -2.746 | 1.191 | -5.081 | -0.411 | -2.3 | 0.022 |
| Intervention vs. Comparison Clinics | -4.757 | 5.013 | -14.582 | 5.069 | -1.0 | 0.343 |
| Intervention Clinics - Pre vs. Post Intervention | -1.945 | 1.558 | -4.998 | 1.108 | -1.3 | 0.212 |

**Figure AF2.2. Fixed Effects Model: Change over Time in the Average Morphine-Equivalent Dose (mg/day) Prescribed per Target Patient.**

In this case (Table AF2.2, Figure AF2.2), both intervention and comparison clinics showed a reduction in the MEDD prescribed per target population patient, with the intervention clinics reducing MEDD by 4.8 mg/day more than the comparison clinics (p=0.34). Although the intervention clinics showed an increase in MEDD by 0.8 mg/day (p=0.47) during the intervention period, they reduced opioid prescribing by 2.7 mg/day (p=0.02) post-intervention.

Other measures were fitted with the mixed effect model, but few of the intervention effects were statistically significant, largely due to the volatility of the monthly outcome measures from prescriber to prescriber, clinic to clinic and over time. Nevertheless, there were some indications of modest relative improvement in the intervention clinic measures versus those of the comparison clinics, e.g., the intervention effect for the “current” treatment agreement rate and the post-intervention effect for average MEDD.

References:

1. McNeish DM, Stapleton LM. The Effect of Small Sample Size on Two-Level Model Estimates: A Review and Illustration. Educational Psychology Review, 2016, 28:295–314.
