# Supplementary material for: Increasing system-wide implementation of opioid prescribing guidelines in primary care: findings from a non-randomized stepped-wedge quality improvement project
Source: BMC Fam Pract. 2020 Nov 28;21:245. doi: 10.1186/s12875-020-01320-9 (PMC7700706; doi:10.1186/s12875-020-01320-9)
Supplement: Supplementary file 3 — Additional file 3. Summary of mixed effects model results [file 12875_2020_1320_MOESM3_ESM.docx]

**Additional File 3. Summary of mixed effects model results**

The tables below summarize the mixed effects modeling (stepped-wedge analysis) results for all measures, with the analyses conducted in the overall target patient population (a) and in the subgroup of target patients treated with high-dose opioids (b).

Results from the primary and secondary outcome measure analyses are presented in Additional File 3 Tables 1-3 (AF3.1- 6).

| **Table AF3.1. Fixed Effects Model: Fraction of Target Patient Population with “Current” Treatment Agreement (Signed in the Past 12 Months).** | | | | | | |
| --- | --- | --- | --- | --- | --- | --- |
| **a. Overall Target Patient Population** | | | | | | |
| **Effect** | **Estimate** | **Standard Error** | **95% CI Lower Bound** | **95% CI Upper Bound** | **t Value** | **Pr > \|t\|** |
| a = Intercept - Intervention clinics | 0.308 | 0.069 | 0.173 | 0.442 | 4.5 | 0.000 |
| a* = Intercept adjust. - Comparison clinics | 0.158 | 0.087 | -0.013 | 0.328 | 1.8 | 0.070 |
| b = Slope - Intervention Clinics | 0.008 | 0.006 | -0.003 | 0.019 | 1.5 | 0.143 |
| b* = Slope adjust. - Comparison clinics | 0.001 | 0.007 | -0.012 | 0.015 | 0.2 | 0.840 |
| c = Intervention Effect | 0.176 | 0.115 | -0.049 | 0.401 | 1.5 | 0.163 |
| d = Post-Intervention Effect | -0.121 | 0.095 | -0.308 | 0.066 | -1.3 | 0.241 |
| Intervention vs. Comparison Clinics | 0.021 | 0.219 | -0.408 | 0.451 | 0.1 | 0.925 |
| Intervention Clinics - Pre vs. Post Intervention | 0.055 | 0.149 | -0.237 | 0.347 | 0.4 | 0.722 |
| **b. Subgroup: Target Patient Population Treated with High-Dose Opioids (≥ 90 mg MED/day)** | | | | | | |
| **Effect** | **Estimate** | **Standard Error** | **95% CI Lower Bound** | **95% CI Upper Bound** | **t Value** | **Pr > \|t\|** |
| a = Intercept - Intervention clinics | 0.364 | 0.052 | 0.262 | 0.466 | 7.0 | <.001 |
| a* = Intercept adjust. - Comparison clinics | 0.194 | 0.069 | 0.058 | 0.330 | 2.8 | 0.005 |
| b = Slope - Intervention Clinics | 0.010 | 0.007 | -0.004 | 0.023 | 1.4 | 0.179 |
| b* = Slope adjust. - Comparison clinics | 0.005 | 0.009 | -0.011 | 0.022 | 0.6 | 0.527 |
| c = Intervention Effect | 0.228 | 0.125 | -0.016 | 0.472 | 1.8 | 0.104 |
| d = Post-Intervention Effect | -0.091 | 0.125 | -0.335 | 0.153 | -0.7 | 0.484 |
| Intervention vs. Comparison Clinics | 0.008 | 0.261 | -0.504 | 0.519 | 0.0 | 0.978 |
| Intervention Clinics - Pre vs. Post Intervention | 0.137 | 0.175 | -0.206 | 0.481 | 0.8 | 0.457 |

| **Table AF3.2. Fixed Effects Model: Fraction of Target Patient Population with “Current” Urine Drug Testing (Completed in the Past 12 Months).** | | | | | | |
| --- | --- | --- | --- | --- | --- | --- |
| **a. Overall Target Patient Population** | | | | | | |
| **Effect** | **Estimate** | **Standard Error** | **95% CI Lower Bound** | **95% CI Upper Bound** | **t Value** | **Pr > \|t\|** |
| a = Intercept - Intervention clinics | 0.265 | 0.052 | 0.163 | 0.368 | 5.1 | <.001 |
| a* = Intercept adjust. - Comparison clinics | 0.077 | 0.068 | -0.056 | 0.211 | 1.1 | 0.255 |
| b = Slope - Intervention Clinics | 0.013 | 0.004 | 0.004 | 0.021 | 2.8 | 0.010 |
| b* = Slope adjust. - Comparison clinics | -0.003 | 0.006 | -0.014 | 0.007 | -0.6 | 0.536 |
| c = Intervention Effect | 0.065 | 0.097 | -0.124 | 0.255 | 0.7 | 0.518 |
| d = Post-Intervention Effect | -0.059 | 0.038 | -0.133 | 0.016 | -1.5 | 0.162 |
| Intervention vs. Comparison Clinics | 0.090 | 0.166 | -0.236 | 0.415 | 0.5 | 0.603 |
| Intervention Clinics - Pre vs. Post Intervention | 0.007 | 0.104 | -0.197 | 0.210 | 0.1 | 0.949 |
| **b. Subgroup: Target Patient Population Treated with High-Dose Opioids (≥ 90 mg MED/day)** | | | | | | |
| **Effect** | **Estimate** | **Standard Error** | **95% CI Lower Bound** | **95% CI Upper Bound** | **t Value** | **Pr > \|t\|** |
| a = Intercept - Intervention clinics | 0.399 | 0.057 | 0.287 | 0.510 | 7.0 | <.001 |
| a* = Intercept adjust. - Comparison clinics | 0.188 | 0.077 | 0.036 | 0.340 | 2.4 | 0.015 |
| b = Slope - Intervention Clinics | 0.011 | 0.005 | 0.001 | 0.021 | 2.2 | 0.039 |
| b* = Slope adjust. - Comparison clinics | -0.008 | 0.006 | -0.020 | 0.005 | -1.2 | 0.235 |
| c = Intervention Effect | 0.143 | 0.080 | -0.014 | 0.301 | 1.8 | 0.113 |
| d = Post-Intervention Effect | -0.110 | 0.096 | -0.299 | 0.078 | -1.2 | 0.284 |
| Intervention vs. Comparison Clinics | 0.217 | 0.188 | -0.152 | 0.585 | 1.2 | 0.283 |
| Intervention Clinics - Pre vs. Post Intervention | 0.033 | 0.124 | -0.211 | 0.276 | 0.3 | 0.798 |

| **Table AF3.3. Fixed Effects Model: Fraction of Target Patient Population with “Current” Depression Screening (Completed in the Past 12 Months).** | | | | | | |
| --- | --- | --- | --- | --- | --- | --- |
| **a. Overall Target Patient Population** | | | | | | |
| **Effect** | **Estimate** | **Standard Error** | **95% CI Lower Bound** | **95% CI Upper Bound** | **t Value** | **Pr > \|t\|** |
| a = Intercept - Intervention clinics | 0.074 | 0.025 | 0.025 | 0.123 | 3.0 | 0.006 |
| a* = Intercept adjust. - Comparison clinics | -0.039 | 0.033 | -0.104 | 0.026 | -1.2 | 0.239 |
| b = Slope - Intervention Clinics | 0.005 | 0.002 | 0.001 | 0.009 | 2.4 | 0.025 |
| b* = Slope adjust. - Comparison clinics | 0.002 | 0.002 | -0.003 | 0.007 | 0.8 | 0.450 |
| c = Intervention Effect | 0.006 | 0.034 | -0.060 | 0.072 | 0.2 | 0.857 |
| d = Post-Intervention Effect | 0.000 | 0.028 | -0.055 | 0.055 | 0.0 | 0.993 |
| Intervention vs. Comparison Clinics | -0.039 | 0.070 | -0.177 | 0.099 | -0.6 | 0.593 |
| Intervention Clinics - Pre vs. Post Intervention | 0.006 | 0.044 | -0.079 | 0.091 | 0.1 | 0.894 |
| **b. Subgroup: Target Patient Population Treated with High-Dose Opioids (≥ 90 mg MED/day)** | | | | | | |
| **Effect** | **Estimate** | **Standard Error** | **95% CI Lower Bound** | **95% CI Upper Bound** | **t Value** | **Pr > \|t\|** |
| a = Intercept - Intervention clinics | 0.052 | 0.029 | -0.005 | 0.110 | 1.8 | 0.087 |
| a* = Intercept adjust. - Comparison clinics | -0.006 | 0.040 | -0.084 | 0.072 | -0.1 | 0.887 |
| b = Slope - Intervention Clinics | 0.010 | 0.003 | 0.004 | 0.015 | 3.6 | 0.001 |
| b* = Slope adjust. - Comparison clinics | -0.003 | 0.003 | -0.010 | 0.004 | -0.9 | 0.385 |
| c = Intervention Effect | 0.007 | 0.041 | -0.074 | 0.088 | 0.2 | 0.871 |
| d = Post-Intervention Effect | -0.006 | 0.060 | -0.123 | 0.112 | -0.1 | 0.929 |
| Intervention vs. Comparison Clinics | 0.066 | 0.095 | -0.120 | 0.252 | 0.7 | 0.504 |
| Intervention Clinics - Pre vs. Post Intervention | 0.001 | 0.072 | -0.139 | 0.142 | 0.0 | 0.985 |

| **Table AF3.4. Fixed Effects Model: Fraction of Target Patient Population with Completed Opioid Misuse Risk Screening** | | | | | | |
| --- | --- | --- | --- | --- | --- | --- |
| **a. Overall Target Patient Population** | | | | | | |
| **Effect** | **Estimate** | **Standard Error** | **95% CI Lower Bound** | **95% CI Upper Bound** | **t Value** | **Pr > \|t\|** |
| a = Intercept - Intervention clinics | -0.001 | 0.016 | -0.032 | 0.030 | -0.1 | 0.963 |
| a* = Intercept adjust. - Comparison clinics | 0.045 | 0.020 | 0.006 | 0.084 | 2.2 | 0.025 |
| b = Slope - Intervention Clinics | 0.001 | 0.002 | -0.002 | 0.004 | 0.6 | 0.573 |
| b* = Slope adjust. - Comparison clinics | 0.002 | 0.002 | -0.002 | 0.006 | 1.0 | 0.299 |
| c = Intervention Effect | 0.036 | 0.020 | -0.003 | 0.075 | 1.8 | 0.110 |
| d = Post-Intervention Effect | 0.007 | 0.010 | -0.012 | 0.025 | 0.7 | 0.513 |
| Intervention vs. Comparison Clinics | -0.011 | 0.054 | -0.117 | 0.096 | -0.2 | 0.849 |
| Intervention Clinics - Pre vs. Post Intervention | 0.043 | 0.022 | -0.001 | 0.086 | 1.9 | 0.091 |
| **b. Subgroup: Target Patient Population Treated with High-Dose Opioids (≥ 90 mg MED/day)** | | | | | | |
| **Effect** | **Estimate** | **Standard Error** | **95% CI Lower Bound** | **95% CI Upper Bound** | **t Value** | **Pr > \|t\|** |
| a = Intercept - Intervention clinics | 0.011 | 0.018 | -0.025 | 0.046 | 0.6 | 0.562 |
| a* = Intercept adjust. - Comparison clinics | 0.044 | 0.024 | -0.003 | 0.091 | 1.8 | 0.068 |
| b = Slope - Intervention Clinics | 0.000 | 0.002 | -0.004 | 0.004 | 0.2 | 0.884 |
| b* = Slope adjust. - Comparison clinics | 0.003 | 0.002 | -0.001 | 0.008 | 1.4 | 0.166 |
| c = Intervention Effect | 0.047 | 0.027 | -0.005 | 0.100 | 1.8 | 0.115 |
| d = Post-Intervention Effect | 0.031 | 0.025 | -0.018 | 0.080 | 1.3 | 0.244 |
| Intervention vs. Comparison Clinics | -0.003 | 0.064 | -0.129 | 0.123 | 0.0 | 0.966 |
| Intervention Clinics - Pre vs. Post Intervention | 0.079 | 0.036 | 0.008 | 0.149 | 2.2 | 0.060 |

| **Table AF3.5. Fixed Effects Model: Fraction of Target Patient Population with PDMP Check Documentation (Completed in the Past 12 Months).** | | | | | | |
| --- | --- | --- | --- | --- | --- | --- |
| **a. Overall Target Patient Population** | | | | | | |
| **Effect** | **Estimate** | **Standard Error** | **95% CI Lower Bound** | **95% CI Upper Bound** | **t Value** | **Pr > \|t\|** |
| a = Intercept - Intervention clinics | -0.034 | 0.028 | -0.090 | 0.021 | -1.2 | 0.234 |
| a* = Intercept adjust. - Comparison clinics | 0.070 | 0.036 | -0.001 | 0.140 | 1.9 | 0.053 |
| b = Slope - Intervention Clinics | 0.017 | 0.008 | 0.002 | 0.032 | 2.2 | 0.036 |
| b* = Slope adjust. - Comparison clinics | 0.005 | 0.009 | -0.013 | 0.024 | 0.6 | 0.571 |
| c = Intervention Effect | 0.348 | 0.184 | -0.013 | 0.709 | 1.9 | 0.096 |
| d = Post-Intervention Effect | 0.273 | 0.198 | -0.115 | 0.662 | 1.4 | 0.240 |
| Intervention vs. Comparison Clinics | 0.494 | 0.344 | -0.179 | 1.167 | 1.4 | 0.224 |
| Intervention Clinics - Pre vs. Post Intervention | 0.621 | 0.272 | 0.089 | 1.154 | 2.3 | 0.084 |
| **b. Subgroup: Target Patient Population Treated with High-Dose Opioids (≥ 90 mg MED/day)** | | | | | | |
| **Effect** | **Estimate** | **Standard Error** | **95% CI Lower Bound** | **95% CI Upper Bound** | **t Value** | **Pr > \|t\|** |
| a = Intercept - Intervention clinics | -0.022 | 0.032 | -0.085 | 0.041 | -0.7 | 0.499 |
| a* = Intercept adjust. - Comparison clinics | 0.089 | 0.041 | 0.007 | 0.170 | 2.1 | 0.032 |
| b = Slope - Intervention Clinics | 0.018 | 0.007 | 0.005 | 0.031 | 2.7 | 0.012 |
| b* = Slope adjust. - Comparison clinics | 0.007 | 0.008 | -0.009 | 0.023 | 0.9 | 0.390 |
| c = Intervention Effect | 0.435 | 0.234 | -0.024 | 0.894 | 1.9 | 0.100 |
| d = Post-Intervention Effect | 0.223 | 0.200 | -0.168 | 0.615 | 1.1 | 0.326 |
| Intervention vs. Comparison Clinics | 0.551 | 0.323 | -0.082 | 1.184 | 1.7 | 0.163 |
| Intervention Clinics - Pre vs. Post Intervention | 0.658 | 0.309 | 0.052 | 1.264 | 2.1 | 0.100 |

| **Table AF3.6. Fixed Effects: Fraction of Target Patient Population Co-prescribed Benzodiazepines (Past 3 Months)** | | | | | | |
| --- | --- | --- | --- | --- | --- | --- |
| **a. Overall Target Patient Population** | | | | | | |
| **Effect** | **Estimate** | **Standard Error** | **95% CI Lower Bound** | **95% CI Upper Bound** | **t Value** | **Pr > \|t\|** |
| a = Intercept - Intervention clinics | 0.174 | 0.012 | 0.151 | 0.197 | 14.9 | <.001 |
| a* = Intercept adjust. - Comparison clinics | 0.058 | 0.017 | 0.025 | 0.091 | 3.5 | 0.001 |
| b = Slope - Intervention Clinics | -0.001 | 0.001 | -0.003 | 0.001 | -0.8 | 0.439 |
| b* = Slope adjust. - Comparison clinics | -0.001 | 0.001 | -0.004 | 0.001 | -1.1 | 0.296 |
| c = Intervention Effect | 0.010 | 0.012 | -0.015 | 0.034 | 0.8 | 0.467 |
| d = Post-Intervention Effect | -0.004 | 0.017 | -0.038 | 0.030 | -0.2 | 0.822 |
| Intervention vs. Comparison Clinics | 0.037 | 0.031 | -0.024 | 0.098 | 1.2 | 0.268 |
| Intervention Clinics - Pre vs. Post Intervention | 0.005 | 0.021 | -0.036 | 0.047 | 0.3 | 0.803 |
| **b. Subgroup: Target Patient Population Treated with High-Dose Opioids (≥ 90 mg MED/day)** | | | | | | |
| **Effect** | **Estimate** | **Standard Error** | **95% CI Lower Bound** | **95% CI Upper Bound** | **t Value** | **Pr > \|t\|** |
| a = Intercept - Intervention clinics | 0.200 | 0.029 | 0.143 | 0.257 | 6.9 | <.001 |
| a* = Intercept adjust. - Comparison clinics | 0.054 | 0.041 | -0.026 | 0.134 | 1.3 | 0.186 |
| b = Slope - Intervention Clinics | 0.000 | 0.002 | -0.003 | 0.003 | -0.1 | 0.943 |
| b* = Slope adjust. - Comparison clinics | -0.002 | 0.002 | -0.005 | 0.002 | -0.8 | 0.432 |
| c = Intervention Effect | -0.003 | 0.025 | -0.052 | 0.047 | -0.1 | 0.923 |
| d = Post-Intervention Effect | -0.020 | 0.023 | -0.065 | 0.025 | -0.9 | 0.413 |
| Intervention vs. Comparison Clinics | 0.015 | 0.044 | -0.071 | 0.102 | 0.3 | 0.740 |
| Intervention Clinics - Pre vs. Post Intervention | -0.022 | 0.034 | -0.088 | 0.043 | -0.7 | 0.525 |

Results from the analyses conducted for other outcomes of interest are presented in Tables AF3.7-AF3.8.

| **Table AF3.7. Fixed Effects Model: Percentage of the Overall Target Patient Population among the Adult Patient Population** | | | | | | |
| --- | --- | --- | --- | --- | --- | --- |
| **Effect** | **Estimate** | **Standard Error** | **95% CI Lower Bound** | **95% CI Upper Bound** | **t Value** | **Pr > \|t\|** |
| a = Intercept - Intervention clinics | 2.04% | 0.26% | 1.53% | 2.56% | 7.7 | <.001 |
| a* = Intercept adjust. - Comparison clinics | -0.11% | 0.31% | -0.72% | 0.51% | -0.3 | 0.737 |
| b = Slope - Intervention Clinics | -0.02% | 0.01% | -0.03% | 0.00% | -2.7 | 0.011 |
| b* = Slope adjust. - Comparison clinics | 0.00% | 0.01% | -0.01% | 0.02% | 0.6 | 0.538 |
| c = Intervention Effect | 0.02% | 0.05% | -0.07% | 0.12% | 0.5 | 0.609 |
| d = Post-Intervention Effect | 0.03% | 0.05% | -0.07% | 0.14% | 0.6 | 0.559 |
| Intervention vs. Comparison Clinics | -0.05% | 0.18% | -0.41% | 0.30% | -0.3 | 0.779 |
| Intervention Clinics - Pre vs. Post Intervention | 0.06% | 0.07% | -0.08% | 0.20% | 0.8 | 0.440 |

| **Table AF3.8. Fixed Effects Model: Change over Time in the Average Morphine-Equivalent Dose (mg/day) Prescribed per Target Patient.** | | | | | | |
| --- | --- | --- | --- | --- | --- | --- |
| **a. Overall Target Patient Population** | | | | | | |
| **Effect** | **Estimate** | **Standard Error** | **95% CI Lower Bound** | **95% CI Upper Bound** | **t Value** | **Pr > \|t\|** |
| a = Intercept - Intervention clinics | 79.253 | 7.659 | 64.242 | 94.264 | 10.4 | <.001 |
| a* = Intercept adjust. - Comparison clinics | -24.926 | 9.400 | -43.349 | -6.503 | -2.7 | 0.008 |
| b = Slope - Intervention Clinics | -0.532 | 0.183 | -0.890 | -0.175 | -2.9 | 0.007 |
| b* = Slope adjust. - Comparison clinics | 0.117 | 0.221 | -0.316 | 0.550 | 0.5 | 0.596 |
| c = Intervention Effect | 0.801 | 1.106 | -1.367 | 2.969 | 0.7 | 0.469 |
| d = Post-Intervention Effect | -2.746 | 1.191 | -5.081 | -0.411 | -2.3 | 0.022 |
| Intervention vs. Comparison Clinics | -4.757 | 5.013 | -14.582 | 5.069 | -1.0 | 0.343 |
| Intervention Clinics - Pre vs. Post Intervention | -1.945 | 1.558 | -4.998 | 1.108 | -1.3 | 0.212 |
| **b. Subgroup: Target Patient Population Treated with High-Dose Opioids (≥ 90 mg MED/day)** | | | | | | |
| **Effect** | **Estimate** | **Standard Error** | **95% CI Lower Bound** | **95% CI Upper Bound** | **t Value** | **Pr > \|t\|** |
| a = Intercept - Intervention clinics | 236.520 | 17.820 | 201.593 | 271.447 | 13.3 | <.001 |
| a* = Intercept adjust. - Comparison clinics | -37.473 | 22.029 | -80.651 | 5.704 | -1.7 | 0.090 |
| b = Slope - Intervention Clinics | -1.669 | 0.634 | -2.912 | -0.426 | -2.6 | 0.014 |
| b* = Slope adjust. - Comparison clinics | 0.489 | 0.777 | -1.034 | 2.012 | 0.6 | 0.529 |
| c = Intervention Effect | 12.428 | 3.927 | 4.730 | 20.125 | 3.2 | 0.002 |
| d = Post-Intervention Effect | -6.505 | 4.320 | -14.972 | 1.962 | -1.5 | 0.133 |
| Intervention vs. Comparison Clinics | -5.815 | 17.586 | -40.282 | 28.653 | -0.3 | 0.741 |
| Intervention Clinics - Pre vs. Post Intervention | 5.923 | 5.646 | -5.143 | 16.989 | 1.1 | 0.295 |
