# Supplementary material for: Increasing system-wide implementation of opioid prescribing guidelines in primary care: findings from a non-randomized stepped-wedge quality improvement project
Source: BMC Fam Pract. 2020 Nov 28;21:245. doi: 10.1186/s12875-020-01320-9 (PMC7700706; doi:10.1186/s12875-020-01320-9)
Supplement: Supplementary file 4 — Additional file 4. Process measures and related findings [file 12875_2020_1320_MOESM4_ESM.docx]

**Additional File 4. Process measures and related findings**

***Process measures*** In addition to outcome measures, which were extracted from the electronic health record, quantitative and qualitative process or explanatory measures were collected from the participating clinicians and clinic staff, and the study team members to better understand the processes underlying the hypothesized change in primary and secondary outcomes.

The process measures, outlined in the prior manuscript,^1^ included the following: 1) Clinician and other clinic staff attendance at the in-person academic detailing and practice facilitation sessions, and completion of the online educational modules. 2) Each educational module completers were given a choice to fill out an anonymous evaluation survey, which used ten questions to rate on a 5-point Likert scale the module’s content, usefulness and quality, and meeting of the learning objectives, and one open-ended question, which gathered qualitative data on anticipated practice changes as a result of participating in the education. 3) Clinician and other clinical staff survey,^1^ developed by the project team, administered in person pre-intervention (at the kick-off meeting) and post-intervention (at the last practice facilitation session). This pre-post survey assessed clinician/staff confidence and attitudes toward the management of patients with opioid-treated chronic pain, using 23 questions with 5-point Likert scale responses and three yes/no questions on the academic detailing presentation’s effectiveness and bias. In addition, the pre-intervention survey included one open-ended question about the anticipated barriers to practice change, and the post-intervention survey asked to rate on a 5-point Likert scale the effectiveness of the practice facilitation (PF) sessions and included 3 open-ended “reflection questions” on clinician/staff experience with the project’s QI process. 4) Practice facilitator and other project team member notes and observations related to the QI initiative implementation at each clinic.

***Intervention participation*** As detailed in the Results section of the manuscript, a total of 215 unique health care providers, including 73 prescribers and 142 other clinic staff from the enrolled 4 family medicine and 5 internal medicine clinics completed at least one component of the QI intervention (QI participants).

***Perceived needs at baseline and after the intervention among clinicians and clinical staff*** Of the 215 unique clinicians/staff who participated in the intervention, 187 returned the pre-intervention survey and 97 returned the post-intervention survey. At baseline (pre-intervention), clinicians and other clinical staff filled out a survey assessing their perceived needs, competencies and practices related to caring for patients with opioid-treated chronic pain. A similar set of questions, which additionally inquired about the self-reported change in these perceptions, was administered at the last PF session (post-intervention survey). Seventy-nine individuals completed both the pre- and post-intervention surveys and were included in the “change” analysis, which contrasted the pre- and post-intervention responses of each individual.

When asked at baseline about the management of patients with opioid-treated chronic pain, the survey responders rated their “current” confidence in management of the target population as “neutral”, and their desire to learn more about such management as “strong,” and expressed wanting to change their current approach to such management (Additional File 4 Table 1, Table AF4.1). After the intervention, the respondents overall reported increased confidence in managing opioid-treated chronic pain, and did not feel the need for additional education/learning or change, with an overall similar pattern of change among prescribers and other clinical staff (Table AF4.1).

**Table AF4.1: Management of patients with opioid-treated chronic pain: perceptions of prescribers and other clinical staff (N=79) before and after the intervention.**

| **Current Management** | **All Responders, N=79** | | | **Prescriber-Responders, N=24** | | | **Other Staff-Responders, N=55** | | |
| --- | --- | --- | --- | --- | --- | --- | --- | --- | --- |
|  | **Pre**  **mean (SD)** | **Post**  **mean (SD)** | ***p* value^2^** | **Pre**  **mean (SD)** | **Post**  **mean (SD)** | ***p* value^2^** | **Pre**  **mean (SD)** | **Post**  **mean (SD)** | ***p* value^2^** |
| **Confidence in Management**^1^ | 3.36 (0.82) | 3.89 (0.79) | 0.000 | 3.46 (0.78) | 3.88 (0.74) | 0.019 | 3.31 (0.85) | 3.90 (0.82) | 0.000 |
| **Desire to Learn More About Management**^1^ | 4.13 (0.92) | 3.55 (0.78) | 0.000 | 4.50 (0.66) | 3.54 (0.72) | 0.000 | 3.96 (0.98) | 3.56 (0.81) | 0.006 |
| **Plan to Change Management**^1^ | 3.77 (0.80) | 3.58 (0.95) | 0.013 | 4.08 (0.88) | 3.83 (0.82) | 0.114 | 3.62 (0.71) | 3.46 (0.99) | 0.031 |

^1^ Response scale: Strongly Disagree (1), Disagree (2), Neutral (3), Agree (4), Strongly Agree (5)

^2^ T-test, one tailed with paired data

When asked at baseline (Table AF4.2) about the frequency of their current use of clinical practices recommended for the monitoring of long-term opioid therapy in chronic pain, the clinicians and clinic staff reported, on average, from “sometimes” to “very often” the utilization of treatment agreements, urine drug testing, and depression screening, as well as applying a shared decision making principles and working together as a team. They rated as less frequent the screening for opioid misuse risk and checking the prescription drug monitoring program (PDMP) database. After the intervention, the self-reported frequency of these practices increased, suggesting a positive change (Table AF4.2).

**Table AF4.2: Frequency of practices related to the monitoring of long-term opioid therapy in chronic pain before and after the intervention among the prescribers and other clinic staff (N=79).**

| **Current Use** | **All Responders, N=79** | | | **Prescribers Responders, N=24** | | | **Other Staff-Responders, N=55** | | |
| --- | --- | --- | --- | --- | --- | --- | --- | --- | --- |
|  | **Pre**  **mean (SD)** | **Post**  **mean (SD)** | ***p* value^2^** | **Pre**  **mean (SD)** | **Post**  **mean (SD)** | ***p* value^2^** | **Pre**  **mean (SD)** | **Post**  **mean (SD)** | ***p* value^2^** |
| **Treatment agreements**^1^ | 3.83 (1.15 ) | 4.11 (1.05) | 0.003 | 4.35 (0.65) | 4.63 (0.65) | 0.035 | 3.50 (1.28) | 3.81 (1.13) | 0.017 |
| **Urine Drug Testing**^1^ | 3.38 (1.19) | 3.79 (1.09) | 0.001 | 3.61 (1.12) | 4.21 (0.69) | 0.001 | 3.26 (1.28) | 3.57 (1.21) | 0.024 |
| **Opioid misuse risk screen**^1^ | 1.92 (1.19) | 2.85 (1.30) | 0.000 | 2.17 (1.27) | 3.04 (1.08) | 0.000 | 1.71 (1.08) | 2.67 (1.49) | 0.001 |
| **Depression screen**^1^ | 3.63 (1.05) | 3.83 (1.12) | 0.017 | 3.46 (0.93) | 4.00 (0.85) | 0.004 | 3.73 (1.12) | 3.73 (1.26) | 0.293 |
| **Check PDMP**^1^ | 2.79 (1.46) | 4.11 (1.06) | 0.000 | 3.08 (1.25) | 4.29 (0.86) | 0.000 | 2.62 (1.57) | 4.00 (1.17) | 0.000 |
| **Apply SDM**^1^ | 3.28 (1.30) | 3.81 (1.05) | 0.001 | 3.67 (1.09) | 4.21 (0.66) | 0.015 | 3.07 (1.37) | 3.56 (1.17) | 0.011 |
| **Work together**^1^ | 3.84 (1.11) | 4.36 (0.75) | 0.000 | 4.04 (1.04) | 4.50 (0.72) | 0.023 | 3.75 (1.14) | 4.30 (0.76) | 0.001 |

PDMP: prescription drug monitoring program; SDM: shared decision making

^1^ Response scale: Never (1), Rarely (2), Sometimes (3), Very Often (4), Extremely Often (5)

^2^ T-test, one tailed with paired data

Prescribers and staff were also asked to evaluate how much change they would like to make in their current practices related to the monitoring of the target population. At baseline, both prescribers and other staff were very interested in changing their existing management practices (Table AF4.3). These responses did not change in a statistically significant way post-intervention, with the exception of opioid misuse risk screen: both the prescribers (p<0.05) and other clinical staff reduced their desire for change in this specific practice (Table AF4.3).

**Table AF4.3: Desire to change the management practices related to long-term opioid therapy in chronic pain before and after the intervention among the clinicians and other staff (N=79).**

| **How Much Change Would You Like to Make?** | **All Responders n=79** | | | **Prescriber Responders n = 24** | | | **Other Staff-Responders n = 55** | | |
| --- | --- | --- | --- | --- | --- | --- | --- | --- | --- |
|  | **Pre**  **mean (SD)** | **Post**  **mean (SD)** | ***p* value^2^** | **Pre**  **mean (SD)** | **Post**  **mean (SD)** | ***p* value^2^** | **Pre**  **mean (SD)** | **Post**  **mean (SD)** | ***p* value^2^** |
| **Treatment Agreements**^1^ | 4.26 (1.59) | 4.51 (1.29) | 0.229 | 4.13 (1.71) | 4.75 (1.33) | 0.087 | 4.34 (1.53) | 4.36 (1.27) | 0.378 |
| **Urine Drug Testing**^1^ | 4.21 (1.62) | 4.37 (1.25) | 0.417 | 4.09 (1.56) | 4.63 (1.10) | 0.115 | 4.29 (1.68) | 4.24 (1.32) | 0.237 |
| **Opioid Misuse Risk Screen**^1^ | 3.94 (1.31) | 3.31 (1.55) | 0.005 | 4.17 (1.23) | 3.17 (1.49) | 0.003 | 3.73 (1.37) | 3.48 (1.63) | 0.318 |
| **Depression Screen**^1^ | 4.19 (1.56) | 4.39 (1.41) | 0.298 | 4.17 (1.33) | 4.25 (1.36) | 0.500 | 4.21 (1.69) | 4.48 (1.46) | 0.258 |
| **Check PDMP**^1^ | 4.19 (1.49) | 4.52 (1.28) | 0.266 | 4.22 (1.45) | 4.58 (1.28) | 0.202 | 4.18 (1.53) | 4.47 (1.29) | 0.460 |
| **Apply SDM**^1^ | 4.12 (1.49) | 4.00 (1.59) | 0.303 | 3.74 (1.63) | 3.96 (1.65) | 0.353 | 4.30 (1.40) | 4.03 (1.58) | 0.185 |
| **Work Together**^1^ | 4.30 (1.54) | 4.51 (1.47) | 0.220 | 4.30 (1.64) | 4.71 (1.40) | 0.200 | 4.30 (1.52) | 4.41 (1.51) | 0.375 |

PDMP: prescription drug monitoring program; SDM: shared decision making

^1^ Response scale: No Change (1), A Little Change (2), Some Change (3), Moderate Change (4), A Great Deal of Change (5), I Already Did This Consistently (6)

^2^ T-test, one tailed with paired data

***Participant satisfaction*** The post-intervention survey administered at the last PF session and the evaluation survey filled out by the completers of online education modules assessed the usefulness of, and satisfaction with, these intervention components.

*The academic detailing* was assessed (“yes/no” questions) as both effective and “appropriate for my practice” by 98% of 189 participants who completed the survey.

*The online spaced education modules* were rated by the module completers on usefulness, quality, appropriateness and effectiveness of the education provided (Table AF4.4). Sixteen of the 68 completers of the opioid-focused module completed the survey; ten of the 50 completers of the shared decision making module completed the survey. The opioid-focused module received high ratings across the evaluated domains, with the shared decision making module rated, on average, as good (Table AF4.4). In addition, participants were asked to provide qualitative comments about anticipated changes because of what they had learned through the online education modules. The majority (75%) of the 16 opioid-focused module respondents anticipated making change in their practice, with the most common change planned (25% of respondents) was to use urine drug testing more regularly with better understanding of results. Thirty percent of the 10 shared decision making module respondents anticipated making change in their practice, with no well-identified qualitative theme in their responses.

**Table AF4.4: Participant Satisfaction with Online Spaced Education Modules.**

| **Module** | **Usefulness**^1^  **mean (SD)** | **Educational Quality**^1^  **mean (SD)** | **Appropriateness**^1^  **mean (SD)** | **Effectiveness**^1^  **mean (SD)** |
| --- | --- | --- | --- | --- |
| **Responsible Opioid Prescribing (N=16)** | 4.06 (0.77) | 4.13 (0.64) | 4.06 (0.77) | 4.06 (0.57) |
| **Shared Decision Making (N=10)** | 2.80 (1.32) | 2.90 (1.45) | 3.56 (0.88) | 3.60 (0.70) |

^1^ Response scale: Poor (1), Fair (2), Good (3), Very Good (4), Excellent (5).

*The practice facilitation component* was highly rated by the 97 responders (Table AF4.5).

**Table AF4.5. Participant Satisfaction with Practice Facilitation (N=97)**

|  | **Addressed Protocol & Process Changes**^1^  **mean (SD)** | **Provided Ongoing Support for Shared Learning**^1^  **mean (SD)** | **Provided Tools & Recommendations for Long-Term**^1^  **mean (SD)** |
| --- | --- | --- | --- |
| **Wave #1 Clinics** | 3.92 (0.80) | 4.04 (0.60) | 4.00 (0.71) |
| **Wave #2 Clinics** | 4.13 (0.58) | 4.03 (0.68) | 3.84 (0.82) |
| **Wave #3 Clinics** | 4.0 (0.59) | 3.89 (0.47) | 3.78 (0.55) |

^1^ Response scale: Strongly Disagree (1), Disagree (2), Neutral (3), Agree (4), Strongly Agree (5).

*Open-ended reflection questions*, administered as a part of the post-intervention survey, gave participants (N=82) an opportunity to provide additional comments on: what improvements in care they experienced; what they learned; and what part of the PF process helped them the most with making change (Table AF4.6). Eighty-two of the survey respondents provided comments, which identified the following main themes in response to these three reflection questions: 1) Overall, five enrolled clinics chose to focus their QI initiatives on general workflow, and four clinics chose to focus on specific areas related to the practices recommended when caring for the target population. The responses to the first reflection question corresponded to these QI focus choices, with clinicians identifying changes in workflows and related processes, and a better application to care of urine drug testing, PDMP checks, and treatment agreements. 2) The respondents identified learning the formal approach to the change process (PDSA model: Plan, Do, Study, Act), gaining confidence in their ability to meaningfully implement change, and the appreciation for team work and their team members as the most important learned lessons from the project. 3) The benefits of an organized approach to change and tracking of its impact, and importance of teamwork were highlighted by the respondents as the most helpful in accomplishing change.

**Table AF4.6. Open-ended reflection questions completed by the prescribers and other clinical staff (N=82) as a part of the post-intervention survey.**

| **Response Themes Per Reflection Question** | **Responses,**  # (%) |
| --- | --- |
| **Question 1: What care improvements occurred?** | |
| Improvement in processes related to pain patients (not one specific process) | 27 (33%) |
| Improvement in urine drug testing | 13 (16%) |
| Increase in check of PDMP | 4 (4%) |
| Increase in use and review of treatment agreement | 7 (9%) |
| Decreased opioid use in pain patient population | 5 (6%) |
| Increase in patient education for pain patients | 3 (4%) |
| Increase in Dire risk assessments | 1 (1%) |
| No change | 8 (10%) |
| No answer | 14 (17%) |
| **Question 2: What have you learned from this process?** | |
| Confidence in change process | 32 (39%) |
| Appreciation for team | 20 (24%) |
| Didn’t learn from PF | 5 (6%) |
| Other | 9 (11%) |
| No answer | 16 (20%) |
| **Question 3: What part of the PF process helped you the most for accomplishing change?** | |
| Addressing Workflows and Process changes | 21 (26%) |
| Understanding team roles | 21 (26%) |
| Facilitated meetings with team | 13 (16%) |
| Outcome data presentation | 10 (12%) |
| Other | 5 (6%) |
| No answer | 11 (13%) |

PF: practice facilitation

References:

1. Zgierska AE, Vidaver RM, Smith P, et al. Enhancing system-wide implementation of opioid prescribing guidelines in primary care: protocol for a stepped-wedge quality improvement project. BMC Health Serv Res. 2018;18(1):415.
